# Relação entre Níveis Plasmáticos Aumentados de Legumain e Propriedades da Placa Aterosclerótica Coronária

**DOI:** 10.36660/abc.20230395

**Published:** 2023-10-23

**Authors:** Yunpeng Deng, Yudong Fan, Di Wu, Zilong Zhang, Miaomiao Zhang, Zhiping Huang, Yuxia Gao

**Affiliations:** 1 Department of Cardiology Tianjin Medical University General Hospital Tianjin China Department of Cardiology, Tianjin Medical University General Hospital, Tianjin – China; 2 Department of Cardiology Emergency General Hospital Beijing China Department of Cardiology, Emergency General Hospital, Beijing – China

**Keywords:** Doença da Artéria Coronariana, Aterosclerose, Biomarcadores

## Abstract

**Fundamento:**

Muitos estudos clínicos confirmaram que a legumain está intimamente relacionada à aterosclerose. Infelizmente, chegaram-se a conclusões diferentes e ainda faltam análises e estudos sobre as características da placa aterosclerótica em pacientes com níveis plasmáticos aumentados de legumain.

**Objetivos:**

Este estudo teve como objetivo investigar a correlação entre as características da legumain e da placa aterosclerótica coronariana.

**Métodos:**

Um total de 81 pacientes com doença cardíaca aterosclerótica coronariana (DCAC), incluindo 43 pacientes com angina instável (AI) e 38 pacientes com angina estável (AE), foram examinados por angiografia coronária. Foi realizado ultrassom intravascular (IVUS) para avaliar as características das placas ateroscleróticas coronarianas, e os níveis plasmáticos de legumain também foram medidos. Valores de p < 0,05 foram considerados significativos.

**Resultados:**

A concentração de legumain foi significativamente maior nos dois subgrupos de doença coronariana do que no grupo controle (todos p<0,001). As concentrações de legumain no grupo AI foram significativamente maiores do que no grupo SA (p=0,001). A área de placa, o índice de remodelamento (IR) e o índice de excentricidade (IE) no grupo AI foram significativamente maiores do que no grupo AE (p<0,001, p=0,001, p=0,001, respectivamente). Houve uma correlação positiva significativa entre os níveis de legumain e IR e IE em pacientes com AI e AE (todos p<0,05).

**Conclusões:**

Níveis plasmáticos elevados de legumain estavam intimamente relacionados com a ocorrência e gravidade da doença coronariana, e as lesões tendiam a ser instáveis. Espera-se que a legumain seja um potencial biomarcador inflamatório para o diagnóstico de doença coronariana e a identificação precoce de lesões coronárias instáveis.

## Figura Central

**Figure:**
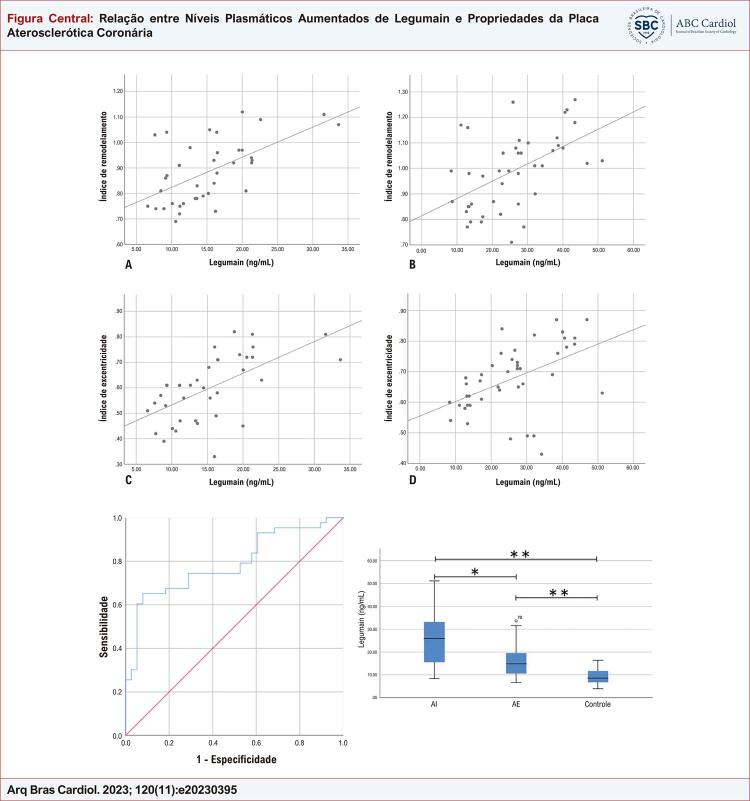
: Relação entre Níveis Plasmáticos Aumentados de Legumain e Propriedades da Placa Aterosclerótica Coronária

## Introdução

Há evidências crescentes de que a inflamação desempenha um papel crucial na formação da aterosclerose, na progressão das placas e na degradação de placas vulneráveis.^1^ Várias células inflamatórias e fatores inflamatórios estão envolvidos na formação da aterosclerose.^2^ As concentrações sanguíneas periféricas desses fatores inflamatórios estão intimamente ligadas à ocorrência e ao prognóstico de eventos cardiovasculares. Fatores inflamatórios como a proteína C reativa (PCR-as) estão significativamente elevados nos níveis sanguíneos periféricos de pacientes com doença coronariana aterosclerótica (DCAC) na fase aguda e têm sido amplamente utilizados na prática clínica.^3^

A legumain, também conhecida como asparagina endopeptidase, é uma cisteína protease lisossomal que desempenha um papel vital na apresentação de antígenos durante a resposta inflamatória.^4^ Recentemente, descobriu-se que ativa proteases como a metaloproteinase de matriz-2, que induz a degradação da matriz extracelular, desempenha um papel importante na formação de aterosclerose e placas vulneráveis, e é provavelmente um potencial preditor de aterosclerose.^5^ Embora estudos sobre legumain e aterosclerose tenham concluído uma forte correlação entre as duas, faltam pesquisas sobre as características das placas ateroscleróticas em pacientes com níveis plasmáticos aumentados de legumain.^6^ Este estudo investigou a correlação entre as características da legumain e da placa aterosclerótica coronariana em pacientes com doença coronariana.^7^

## Métodos

### População do estudo

Foram selecionados 81 pacientes, na faixa etária de 35 a 80 anos, incluindo 38 homens e 43 mulheres, internados no Hospital Geral de Emergência com diagnóstico previsto de doença coronariana entre setembro de 2021 e outubro de 2022. Todos foram confirmados como portadores de doença coronariana por angiografia coronária, e foi realizada uma ultrassonografia intravascular (IVUS). Esses pacientes foram diagnosticados com angina estável (AE, 38 casos) ou angina instável (AI, 43 casos) e apresentavam estenose da artéria coronária com redução de pelo menos 50% no diâmetro do lúmen e pelo menos 2,25 mm de diâmetro na angiografia coronariana.^8^ Trinta e sete indivíduos correspondentes às características de gênero e idade dos grupos experimentais foram inscritos como controles saudáveis. O consentimento informado por escrito foi obtido de todos os pacientes antes do estudo. O protocolo do estudo concordou com as diretrizes aprovadas pelo comitê de ética de nossa instituição. (Número de aprovação ética: K22-6; Número do ensaio clínico: ChiCTR2200058185).

AE foi definida como nenhuma alteração na frequência, duração ou intensidade dos sintomas anginosos com enzimas cardíacas normais nas últimas 6 semanas. AI foi definida como 1) angina de repouso, 2) novo início de angina acelerada nos últimos 2 meses, ou 3) angina acelerada, mas angina crônica estável em pacientes que não tiveram angina de repouso nos 2 meses anteriores.^9^ Os controles saudáveis foram aqueles pacientes sem achados coronarianos anormais. Os critérios de exclusão foram infarto agudo do miocárdio (IAM), lesões oclusivas crônicas, lesões calcificadas ou difusas, doença infecciosa ou autoimune, intervenção coronária percutânea prévia ou cirurgia de revascularização miocárdica, valvopatia, insuficiência cardíaca congestiva (FEVE < 40%), malignidade, doença hematológica, doença renal (creatinina plasmática ≥ 2,2 mg/dL) e doença hepática grave (níveis plasmáticos de alanina transaminase >120 U/L).

### Medição de legumain plasmática

O sangue venoso foi coletado de todos os indivíduos em jejum. O plasma foi extraído e armazenado a -80°C. As concentrações plasmáticas de legumain foram quantificadas utilizando o Quality ELISA Assay Kit (Elisa Biotech Systems, Shanghai, PR China). Os intervalos de detecção dos ELISAs usados para medir legumain foram de 2,5 ng/mL a 80 ng/mL. O coeficiente de variação intraensaio (%) e o coeficiente de variação interensaio (%) foram < 15%.^10^ Além disso, PCR-as, homocisteína, lipoproteína de baixa densidade (LDL-c) e outros exames laboratoriais foram concluídos simultaneamente.

### Exame de ultrassom intravascular (IVUS)

Após a conclusão da angiografia coronária, foi realizado exame de IVUS nos principais vasos afetados. O exame foi realizado utilizando aparelho IVUS da Boston Scientific e equipamento acompanhante.^11^ O cateter do IVUS foi enviado distalmente ao vaso alvo e avançou até o segmento proximal a uma velocidade de 0,5 mm/s por meio de dispositivo de retração automático.^12^ As imagens de IVUS foram continuamente registradas e analisadas quantitativamente por dois investigadores independentes e experientes em IVUS, cegos para este estudo usando software. Os segmentos de referência proximais e distais foram as seções transversais de aparência mais normal dentro de 10 mm distais e proximais à lesão, sem qualquer ramo lateral significativo. A membrana elástica externa (MEE) e a área de secção transversal do lúmen (AST) foram medidas e, em seguida, a AST da placa foi calculada (AST da MEE - AST do lúmen). Carga de placa = (AST do MEE - AST do lúmen)/AST do MEE multiplicado por 100%.^13^ De acordo com a maior espessura de placa e a espessura mínima de placa, foi calculado o índice excêntrico de placa (IE) (espessura máxima de placa - espessura mínima de placa/espessura máxima de placa) multiplicado por 100%. IE<0,5 foi considerado placa concêntrica; IE≥0,5 foi considerado placa excêntrica. O índice de remodelamento (IR) é definido como a AST da membrana de remodelamento da lesão dividida pela média da AST do segmento de referência. Geralmente, IR>1,05 indica remodelamento positivo e IR<0,95 indica remodelamento negativo.^14^

### Análise estatística

A análise estatística foi realizada utilizando o pacote de software SPSS 25. O teste de Kolmogorov-Smirnov testou a normalidade das variáveis contínuas. Variáveis contínuas com distribuição normal são apresentadas como média±desvio padrão(DP) e comparadas pelo teste t de Student não pareado ou análise de variância unidirecional (ANOVA). Não houve mais utilização de testes post hoc na ANOVA, uma vez que a variável relevante não era o indicador principal. Variáveis com distribuição não normal foram representadas como medianas e intervalos interquartis e comparadas usando o teste U de Mann-Whitney ou o teste de Kruskal-Wallis (teste KW). O método Bonferroni foi usado para post hoc análise no teste KW. As variáveis categóricas foram expressas como frequências e porcentagens e comparadas pelo teste do qui-quadrado ou pelo método exato de probabilidade de Fisher.

As correlações entre legumain e variáveis como IR e IE foram avaliadas pela análise de correlação de Pearson ou Spearman. O valor diagnóstico da legumain para AI foi avaliado por meio de curvas características de operação do receptor (ROC). Os fatores de risco cardiovascular para AI foram avaliados por meio de análise de regressão logística múltipla. Um valor p bilateral de 0,05 foi considerado estatisticamente significativo.

## Resultados

### Informações e características de base

As informações iniciais e as características de todos os pacientes estão resumidas na Tabela 1. Houve diferenças significativas entre os três grupos nos níveis séricos de LDL-c e usuários de estatinas (%).

**Tabela 1:** – Características demográficas e bioquímicas de todos os participantes

Característica	AI (n=43)	AE (n=38)	Controle (n=37)	p
Idade, anos	67,0(57,0,71,0)	67,0(52,8,73,0)	66,0(56,0,73,0)	0,624
Masculino, n (%)	22(51,2)	16(42,1)	18(48,6)	0,707
IMC (kg/m^2^)	23h40(20h70,27h10)	24,35(20,23,26,33)	25,00(22,60,26,95)	0,361
Hipertensão, n (%)	22(51,2)	16(42,1)	16(43,2)	0,669
Diabetes mellitus, n (%)	20(46,5)	12(31,6)	9(24,3)	0,102
Tabagismo, n (%)	18(41,9)	17(44,7)	15(40,5)	0,931
Álcool (%)	21(48,8)	14(36,8)	14(37,8)	0,473
LDL-C (mmol/L)	2,72±0,98	2,53±0,87	2,18±0,73	0,024
HDL-C (mmol/L)	1,04(0,86,1,41)	1,02(0,85,1,24)	0,98(0,77,1,11)	0,431
Homocisteína (μmol/L)	13.04(11.23,16.44)	14.10(12.17,16.85)	13,98(11,76,15,71)	0,480
PCRas (mg/L)	1,03(0,66,1,78)	1,03(0,59,1,59)	0,98(0,57,1,92)	0,856
Glicose de jejum (mmol/L)	6,12(5,57,7,56)	5,91(5,23,7,14)	6.02(5.17,7.21)	0,483
Triglicerídeo (mmol/L)	1,98(1,30,2,40)	1,87(1,38,2,67)	1,47(1,22,2,14)	0,342
Colesterol (mmol/L) Usuário de estatina, n (%)	4,15(3,59,5,01)29(67,4)	3,99(3,64,5,18)27(71,1)	3,99(3,59,4,73)15(40,5)	0,873 0,012

As variáveis contínuas são apresentadas como médias ± DP, mediana (intervalo interquartil). Variáveis categóricas foram expressas como frequências e porcentagens. AI: angina instável; AE: angina estável; IMC: índice de massa corporal; LDL-C: lipoproteína de baixa densidade; HDL-C: lipoproteína de alta densidade; PCRas: proteína C reativa de alta sensibilidade.

### Níveis plasmáticos de legumain em todas as populações do estudo

**Figura 1:**
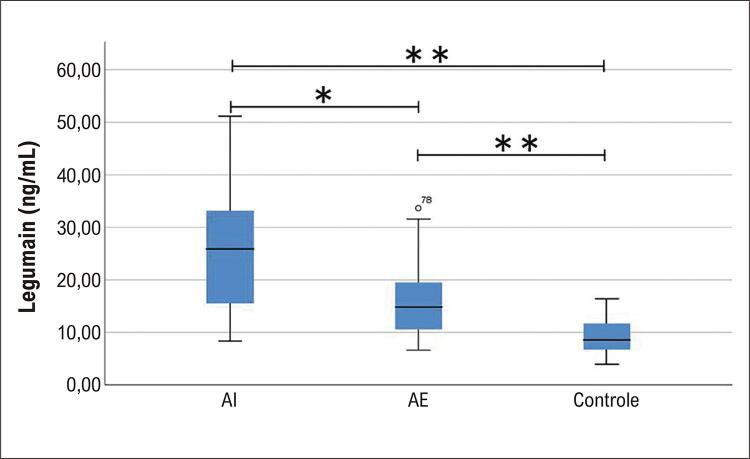
– Comparações dos níveis de legumain entre os três grupos.

AI: angina instável; AE: angina estável; *p<0,01, **p<0,001.

Os níveis de legumain nos três grupos foram 25,90 [14,11, 34,19] ng/ml, 14,83 [10,45, 19,64] ng/ml e 8,55[6,70,11,81] ng/ml, respectivamente. As diferenças entre os grupos AI e AE, entre os grupos AI e controle e entre os grupos AE e controle foram estatisticamente significativas (p=0,001, p<0,001, p<0,001, respectivamente; Figura 1).

### Parâmetros do IVUS

Não houve diferenças entre os dois grupos em relação ao comprimento da lesão, diâmetro mínimo do lúmen (DLM), área mínima do lúmen (AML) ou carga de placa. A área de placa, IR e IE no grupo AI foram significativamente maiores do que no grupo AE (p<0,001, p=0,001, p=0,003, respectivamente, mostrados na Tabela 2).

**Tabela 2:** – Parâmetros de IVUS em pacientes com AI versus pacientes com AE

Variáveis	AI (n=43)	AE (n=38)	p
Comprimento da lesão, mm	31,77±8,80	31,60±7,86	0,930
DML, mm	1,47(1,33,1,67)	1,44(1,31,1,67)	0,716
AML, mm^2^	2,66(2,38,2,95)	2,62(2,09,3,02)	0,936
Área da placa, mm^2^	13,25±2,19	11,06±1,95	0,000
Carga de placa, %	76,66(71,12,81,29)	73,61(69,44,78,77)	0,195
Índice de remodelamento	0,99±0,15	0,89±0,12	0,001
Remodelamento positivo[n (%)]	17(39,5)	4(10,5)	0,003
Remodelamento negativo [n (%)]	16(37,2)	26(68,4)	0,005
Índice de excentricidade	0,68±0,11	0,60±0,13	0,003
Lesão excêntrica [n (%)]	39(90,7)	28(73,7)	0,043
Lesão concêntrica [n (%)]	4(9,3)	10(26,3)	0,043

As variáveis contínuas são apresentadas como médias ± DP, mediana (intervalo interquartil). Variáveis categóricas foram expressas como frequências e porcentagens. AI: angina instável; AE: angina estável; DML: diâmetro mínimo do lúmen; AML: área mínima do lúmen.

### Correlações da concentração de legumain com parâmetros de IVUS

**Figura 2:**
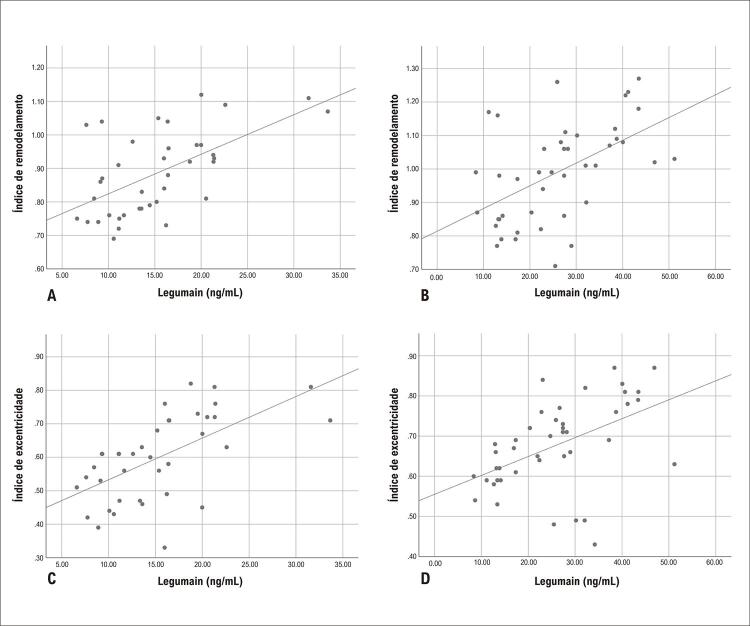
– Correlações da concentração de legumain com índice de remodelamento (A,B) índice de excentricidade (C,D) nos grupos AE e AI. AE: angina estável; AI: angina instável.

Os coeficientes de correlação entre concentração de legumain e IR nos grupos AI e AE foram 0,523 e 0,553, respectivamente (todos p<0,001). Os coeficientes de correlação entre concentração de legumain e IE nos grupos AI e AE foram 0,486 (p=0,001) e 0,651 (p<0,001), respectivamente. Os dados acima indicaram uma correlação positiva significativa entre legumain e RI e entre legumain e placa IE em ambos os grupos AI e AE (mostrados na Figura 2).

### Análise de regressão logística múltipla de fatores de risco cardiovascular para pacientes com AI

Para investigar fatores de risco cardiovascular em pacientes com AI, após incluir fatores de risco cardiovascular tradicionais, como tabagismo e LDL-C, realizamos análises de regressão logística múltipla juntamente com parâmetros de IVUS que diferiram entre os grupos AI e AE. A análise de regressão mostrou que os fatores de risco cardiovascular independentes para pacientes com AI foram concentração de legumain e área de placa coronariana (Tabela 3).

**Tabela 3:** – Análise de regressão logística múltipla dos fatores de risco cardiovascular para os pacientes com AI

	Razão de probabilidade	(IC 95%)	Valor p
AI			
Concentração de legumain	1.198	1.058-1.356	0,004
Área de placa coronária	1.652	1.150-2.375	0,007

A variável dependente foi a presença de AI. As variáveis covariáveis incluíram idade, sexo, hipertensão, tabagismo ou não, diabetes mellitus, IMC, homocisteína, LDL-Cl, glicemia de jejum, triglicerídeos, colesterol total, concentração de legumain, IR, IE, e área de placa. AI: angina instável.

### Legumain como fator de diagnóstico de AI

**Figura 3:**
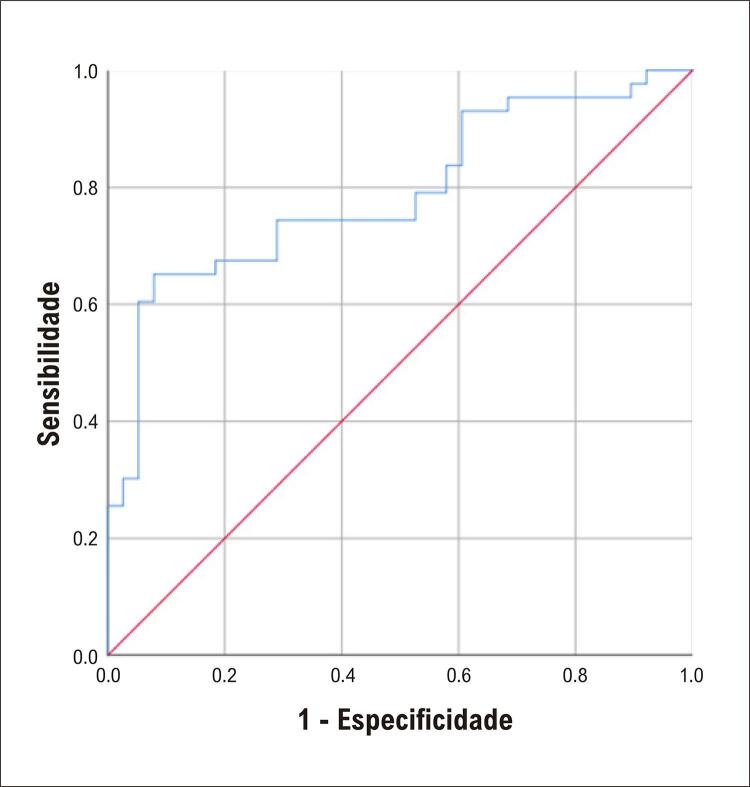
– Curvas ROC (Receiver Operating Characteristic) da legumain para diagnóstico de AI. A área sob a curva da legumain foi de 0,789 (intervalo de confiança [IC] de 95%, 0,689–0,888, p<0,001).

A análise ROC foi realizada para determinar a sensibilidade e especificidade dos níveis de legumain no diagnóstico de AI. A área sob a curva ROC (AUC) dos níveis de legumain para o diagnóstico de AI foi de 0,789 (IC 95%: 0,689-0,888, p<0,001). A análise ROC mostrou que o valor de corte ideal do nível de legumain foi de 21,68 ng/mL, e a sensibilidade e especificidade para o diagnóstico de AI foram de 65,1% e 92,1%, respectivamente. Estes resultados sugerem que a legumain está altamente correlacionada com lesões instáveis. Espera-se que seja um fator inflamatório confiável para o diagnóstico de doença coronariana, especialmente AI, para orientar a detecção clínica precoce de pessoas com alto risco de lesões nas artérias coronárias (mostradas na Figura 3).

## Discussão

A aterosclerose coronariana é uma doença sistêmica com mecanismos específicos, complexos e ainda não totalmente compreendidos.^15^ Entre eles, os fatores inflamatórios desempenham um papel crucial na progressão da aterosclerose coronariana e na formação de placas instáveis, o que foi confirmado em muitos estudos anteriores.^16^

Como fator inflamatório, a legumain está envolvida na apresentação de antígenos durante a aterosclerose e pode induzir a formação e progressão de placas instáveis, como foi provisoriamente demonstrado em muitos estudos. Já em 2006, Papaspyridonos et al. identificaram um aumento significativo na expressão do gene legumain em placas ateroscleróticas instáveis.^17^ Em 2020, Hui Yang et al. investigaram a associação da legumain com infarto agudo do miocárdio e concluíram que a legumain é um preditor de mortalidade por todas as causas e um potencial alvo terapêutico no infarto agudo do miocárdio.^18^ Ao mesmo tempo, Lunde et al. cheguei quase à conclusão oposta.^19^ Eles concluíram que a legumain é regulada positivamente em eventos cardiovasculares agudos e está associada a melhores resultados. As razões para os diferentes resultados de experiências anteriores podem ser as seguintes: Existe uma interação complexa entre legumain e macrófagos M1 e M2, de modo que os efeitos anti-inflamatórios e pró-inflamatórios dos macrófagos exercem diferentes graus de influência em diferentes fases da doença. e outros fatores externos.^20^ Além disso, a função plaquetária está associada à liberação de legumain, e uma alta proporção de pacientes com doenças cardiovasculares utiliza medicamentos antiplaquetários como a aspirina, um fenômeno que também pode influenciar os resultados do estudo.^21^ Nossos resultados mostraram que os níveis de legumain foram significativamente maiores em pacientes com AI e AE do que em controles saudáveis, enquanto as concentrações de legumain no grupo de AI foram significativamente maiores do que aquelas em indivíduos com AE, sugerindo que níveis mais elevados de legumain estão associados ao desenvolvimento de doença aterosclerótica coronariana e a formação de lesões instáveis.

Neste estudo, não encontramos diferenças significativas no comprimento da lesão, DLM e AML comparando os parâmetros do IVUS dos vasos da lesão coronariana em pacientes dos grupos AI e AE. Notavelmente, a área da placa, o IR coronário e o IE da placa foram significativamente maiores no grupo AI do que no grupo AE. Além disso, a incidência de remodelamento positivo e placa excêntrica foi significativamente maior no grupo AI do que no grupo AE, e a diferença foi estatisticamente significativa. Isto também indicou que a gravidade e o risco da doença coronariana estão intimamente relacionados com a instabilidade da lesão, mas não necessariamente relacionados diretamente com a taxa de estenose dos vasos coronários. Em geral, as placas excêntricas são mais vulneráveis que as placas concêntricas e têm maior probabilidade de romper sob vários estresses intraluminais, que são mais frequentemente encontrados em segmentos de lesão instáveis.^22^ Além disso, o remodelamento positivo está amplamente presente nos segmentos de lesão aterosclerótica. De acordo com estudos anteriores de IVUS, as alterações vasculares na síndrome coronariana aguda são propensas a remodelamento positivo em comparação com lesões vasculares AE, e o remodelamento positivo está intimamente associado a eventos adversos, como ruptura de placa e trombose.^23^ Além disso, descobrimos que a legumain foi significativamente correlacionada positivamente com o IR e o IE da placa nos grupos AI e AE. Em resumo, o risco de desenvolver doença cardiovascular aterosclerótica aumenta em pessoas sem doença coronariana à medida que os níveis de legumain aumentam. Na população com doença coronariana, níveis mais elevados de legumain indicam lesões vasculares mais graves e placas mais instáveis.

Muitos fatores inflamatórios estão associados à aterosclerose, mas poucos apresentam alta especificidade e podem ser amplamente utilizados na prática clínica. Por exemplo, fatores inflamatórios tradicionais, como PCR-as e homocisteína, têm muitos fatores de influência, incluindo baixa especificidade e aplicação clínica limitada. O fator inflamatório legumain estudado neste estudo está altamente correlacionado com a doença aterosclerótica coronariana e é atualmente um tema de pesquisa importante por estudiosos. A análise de regressão logística multivariada sobre fatores de risco cardiovascular em pacientes com AI neste estudo também sugeriu ainda que o nível plasmático de legumain era um fator de risco cardiovascular independente em pacientes com AI (OR = 1,198, p = 0,004). A AUC para o diagnóstico de AI com o nível de legumain neste estudo atingiu 0,789, e a sensibilidade e a especificidade de um nível de legumain de 21,68 ng/mL para o diagnóstico de AI foram de 65,1% e 92,1%, respectivamente. Os resultados acima sugerem a viabilidade, precisão e especificidade do legumain para o diagnóstico de AI. Pode-se dizer que se prevê que a legumain se torne um fator inflamatório para o diagnóstico da doença aterosclerótica coronariana.^24^

Devido à falta de estudos anteriores e relatos na literatura sobre a concentração de legumain em pacientes com doença arterial coronariana e AI, o tamanho da amostra foi determinado por um estudo piloto neste estudo. A partir dos pré-experimentos, concluímos que a concentração de legumain para os três grupos de indivíduos foi de 21,18±12,28 ng/ml no grupo AI, 14,94±6,14 ng/ml no grupo AE e 8,75±3,01 ng/ml no grupo controle. O tamanho geral da amostra foi calculado em 99 casos (33 casos por grupo) usando o software PASS, e o tamanho final da amostra foi determinado em 118 casos, considerando a perda de acompanhamento e dados incompletos.

Nosso estudo tem várias limitações. Primeiro, este é um estudo de caso-controle, unicêntrico, não randomizado, com amostra pequena, falta de acompanhamento e incapacidade de explicar a causalidade. Em segundo lugar, o IVUS utilizado neste estudo não tinha capacidade de VH-IVUS, por isso foi impossível classificar as lesões com mais precisão.^25^ Terceiro, um subgrupo de pacientes com IAM não foi estabelecido neste estudo porque a realização de IVUS prolonga o tempo de operação e pode afetar o prognóstico dos pacientes. Finalmente, o IVUS tem resolução limitada na identificação de lesões coronárias e é menos capaz de identificar algumas lesões específicas do que a OCT.^26^

## Conclusões

Altos níveis plasmáticos de legumain estão intimamente associados ao desenvolvimento de doença aterosclerótica coronariana e à progressão de lesões instáveis. A detecção da legumain plasmática pode ajudar a identificar mais cedo pacientes com alto risco de doença coronariana, para que a intervenção possa ocorrer mais cedo e o prognóstico possa ser melhorado.
