# Supplementary material for: Relação entre Níveis Plasmáticos Aumentados de Legumain e Propriedades da Placa Aterosclerótica Coronária
Source: Arq Bras Cardiol. 2023 Oct 23;120(11):e20230395. [Article in Portuguese] doi: 10.36660/abc.20230395 (PMC10593388; doi:10.36660/abc.20230395)
Supplement: Translation

# Translation: Relationship between Increased Plasma Levels of Legumain and Properties of Coronary Atherosclerotic Plaque

**Authors:** Yunpeng Deng, Yudong Fan, Di Wu, Zilong Zhang, Miaomiao Zhang, Zhiping Huang, Yuxia Gao

**Affiliations:** 1 China Department of Cardiology, Tianjin Medical University General Hospital, Tianjin – China; 2 China Department of Cardiology, Emergency General Hospital, Beijing – China

**Keywords:** Coronary Artery Disease, Atherosclerosis, Biomarkers

## Abstract

**Background:**

Many clinical studies have confirmed that legumain is closely related to atherosclerosis. Unfortunately, different conclusions have been reached, and analyses and studies on atherosclerotic plaque characteristics in patients with increased plasma levels of legumain are still lacking.

**Objectives:**

This study aimed to investigate the correlation between legumain and coronary atherosclerotic plaque characteristics.

**Methods:**

A total of 81 patients with coronary atherosclerotic heart disease (CHD), including 43 patients with unstable angina (UA) and 38 patients with stable angina (SA), were screened by coronary angiography. Intravascular ultrasound (IVUS) was performed to evaluate the characteristics of coronary atherosclerotic plaques, and plasma legumain levels were also measured. Values of p < 0.05 were considered significant.

**Results:**

Legumain concentration was significantly higher in the two CHD subgroups than in the control group (all p<0.001). Legumain concentrations in the UA group were significantly higher than in the SA group (p=0.001). The plaque area, remodeling index (RI), and eccentricity index (EI) in the UA group were significantly higher than those in the SA group (p<0.001, p=0.001, p=0.001, respectively). There was a significant positive correlation between legumain levels and RI and EI in both UA and SA patients (all p<0.05).

**Conclusions:**

High plasma levels of legumain were closely related to the occurrence and severity of CHD, and the lesions tended to be unstable. Legumain is expected to be a potential inflammatory biomarker for the diagnosis of CHD and the early identification of unstable coronary lesions.

## Introduction

There is growing evidence that inflammation plays a crucial role in the formation of atherosclerosis, the progression of plaques, and the breakdown of vulnerable plaques.^1^ Various inflammatory cells and inflammatory factors are involved in atherosclerosis formation.^2^ The peripheral blood concentrations of these inflammatory factors are closely linked to the occurrence and prognosis of cardiovascular events. Inflammatory factors such as C-reactive protein (hs-CRP) are significantly elevated in peripheral blood levels of patients with coronary atherosclerotic heart disease (CHD) in the acute phase and have been widely used in clinical practice.^3^

Legumain, also known as asparagine endopeptidase, is a lysosomal cysteine protease that plays a vital role in antigen presentation during the inflammatory response.^4^ Recently, it has been found to activate proteases such as matrix metalloproteinase-2, which induces extracellular matrix degradation, plays an important role in forming atherosclerosis and vulnerable plaques, and is most likely a potential atherosclerosis predictor.^5^ Although studies on legumain and atherosclerosis have concluded a strong correlation between the two, there is a lack of research on the characteristics of atherosclerotic plaques in patients with increased plasma levels of legumain.^6^ This study investigated the correlation between legumain and coronary atherosclerotic plaque characteristics in patients with CHD.^7^

## Methods

### Study population

A total of 81 patients, age range 35~80 years, including 38 males and 43 females, admitted to the Emergency General Hospital with a predicted diagnosis of CHD between September 2021 and October 2022 were selected. All were confirmed to have CHD by coronary angiography, and an intravascular ultrasound (IVUS) was also performed. These patients were diagnosed with stable angina (SA, 38 cases) or unstable angina (UA, 43 cases) and presented with coronary artery stenosis with at least 50% reduction in lumen diameter stenosis and at least 2.25 mm in diameter on coronary angiography.^8^ Thirty-seven subjects matching the gender and age characteristics of the experimental groups were enrolled as healthy controls. Written informed consent was obtained from all patients before the study. The study protocol agreed with the guidelines approved by the ethics committee at our institution. (Ethics Approval Number: K22-6;Clinical Trial Number: ChiCTR2200058185)

SA was defined as no change in frequency, duration, or intensity of anginal symptoms with normal cardiac enzymes in the past 6 weeks. UA was defined as 1) resting angina, 2) new onset accelerated angina within the past 2 months, or 3) accelerated angina but chronic stable angina in patients who had not had resting angina in the previous 2 months.^9^ Healthy controls were those patients without abnormal coronary findings. Exclusion criteria were acute myocardial infarction(AMI), chronic occlusive lesions, calcified or diffuse lesions, infectious or autoimmune disease, prior percutaneous coronary intervention or coronary artery bypass grafting, valvular heart disease, congestive heart failure (LVEF < 40%), malignancy, hematologic disease, renal disease (plasma creatinine ≥ 2.2 mg/dL), and severe liver disease (plasma alanine transaminase levels >120 U/L).

### Measurement of plasma legumain

Venous blood was drawn from all subjects in a fasting state. Plasma was extracted and stored at -80 °C. Plasma legumain concentrations were quantified using the Quality ELISA Assay Kit (Elisa Biotech Systems, Shanghai, PR China). The detection ranges of the ELISAs used to measure legumain were 2.5 ng/mL-80 ng/mL. The intra-assay coefficient of variation (%) and interassay coefficient of variation (%) were < 15%.^10^ In addition, hs-CRP, homocysteine, low-density lipoprotein (LDL-c), and other laboratory tests were completed simultaneously.

### Intravascular ultrasound (IVUS) examination

After the completion of coronary angiography, an IVUS examination was performed on the main diseased vessels. The examination was performed using Boston Scientific’s IVUS machine and accompanying equipment.^11^ The IVUS catheter was sent distal to the target vessel and advanced to the proximal segment at a speed of 0.5 mm/s using an automatic pullback device.^12^ The IVUS images were continuously recorded and quantitatively analyzed using software by two independent, experienced IVUS investigators blinded to this study. Proximal and distal reference segments were the most normal-looking cross-sections within 10 mm distal and proximal to the lesion without any significant side branch. The external elastic membrane (EEM) and lumen cross-sectional area (CSA) were measured, and then the plaque CSA was calculated (EEM CSA - lumen CSA). Plaque burden = (EEM CSA - lumen CSA)/EEM CSA multiplied by 100%.^13^ According to the largest plaque thickness and minimum plaque thickness, the plaque eccentric index (EI) was calculated (maximum plaque thickness - minimum plaque thickness/maximum plaque thickness) multiplied by 100%. EI<0.5 was considered concentric plaque; EI≥0.5 was considered eccentric plaque. The remodeling index (RI) is defined as the remodeling membrane CSA of the lesion divided by the mean CSA of the reference segment. Generally, RI>1.05 indicates positive remodeling, and RI<0.95 indicates negative remodeling.^14^

### Statistical analysis

Statistical analysis was performed using the SPSS 25 software package. The Kolmogorov-Smirnov test tested the normality of continuous variables. Continuous variables with normal distributions are shown as the mean±standard deviation(SD) and were compared by Student’s unpaired t-test or one-way analysis of variance (ANOVA). There was no further use of post hoc tests in the ANOVA since the relevant variable was not the main indicator. Nonnormally distributed variables were represented as medians and interquartile ranges and were compared using the Mann–Whitney U-test or the Kruskal–Wallis test(K-W test). The Bonferroni method was used for post hoc analysis in the K-W test. Categorical variables were expressed as frequencies and percentages and were compared using the chi-square test or Fisher’s exact probability method.

Correlations between legumain and variables such as RI and EI were evaluated using Pearson or Spearman correlation analysis. The diagnostic value of legumain for UA was evaluated using receiver operating characteristic (ROC) curves. Cardiovascular risk factors for UA were evaluated using multiple logistic regression analysis. A two-sided p-value of 0.05 was considered statistically significant.

## Results

### Baseline information and characteristics

The baseline information and characteristics of all patients are summarized in Table 1. There were significant differences among the three groups in serum LDL-c levels and statin users (%).

**Table 1:** – Demographic and biochemical characteristics of all participants

Characteristic	UA(n=43)	SA(n=38)	Control(n=37)	p
Age, yrs	67.0(57.0,71.0)	67.0(52.8,73.0)	66.0(56.0,73.0)	0.624
Male, n(%)	22(51.2)	16(42.1)	18(48.6)	0.707
BMI (kg/m^2^)	23.40(20.70,27.10)	24.35(20.23,26.33)	25.00(22.60,26.95)	0.361
Hypertension, n (%)	22(51.2)	16(42.1)	16(43.2)	0.669
Diabetes mellitus, n (%)	20(46.5)	12(31.6)	9(24.3)	0.102
Smoking, n (%)	18(41.9)	17(44.7)	15(40.5)	0.931
Drinking, n (%)	21(48.8)	14(36.8)	14(37.8)	0.473
LDL-C (mmol/L)	2.72±0.98	2.53±0.87	2.18±0.73	0.024
HDL-C (mmol/L)	1.04(0.86,1.41)	1.02(0.85,1.24)	0.98(0.77,1.11)	0.431
Homocystein(μmol/L)	13.04(11.23,16.44)	14.10(12.17,16.85)	13.98(11.76,15.71)	0.480
hs-CRP (mg/L)	1.03(0.66,1.78)	1.03(0.59,1.59)	0.98(0.57,1.92)	0.856
Fasting glucose (mmol/L)	6.12(5.57,7.56)	5.91(5.23,7.14)	6.02(5.17,7.21)	0.483
Triglyceride (mmol/L)	1.98(1.30,2.40)	1.87(1.38,2.67)	1.47(1.22,2.14)	0.342
Cholesterol (mmol/L) Statin user, n (%)	4.15(3.59,5.01)29(67.4)	3.99(3.64,5.18)27(71.1)	3.99(3.59,4.73)15(40.5)	0.873 0.012

Continuous variables are presented as means ± SD, median (interquartile range). Categorical variables were expressed as frequencies and percentages. UA: unstable angina; SA: stable angina; BMI: body index mass; LDL-C: low-density lipoprotein cholesterol; HDL-C: high-density lipoprotein cholesterol; hs-CRP: high-sensitivity C-reactive protein.

### Plasma levels of Legumain in all study populations

Legumain levels in the three groups were 25.90 [14.11, 34.19] ng/ml, 14.83 [10.45, 19.64] ng/ml, and 8.55[6.70,11.81] ng/ml, respectively. The differences between the UA and SA groups, between the UA and control groups, and between the SA and control groups were statistically significant (p=0.001, p<0.001, p<0.001, respectively; Figure 1).

**Figure 1:** – Comparisons of the legumain levels among the three groups.

UA: unstable angina; SA: stable angina;*p<0.01, **p<0.001).

### Parameters of IVUS

There were no differences between the two groups concerning lesion length, minimum lumen diameter (MLD), minimum lumen area (MLA), or plaque burden. The plaque area, RI, and EI in the UA group were significantly higher than in the SA group (p<0.001, p=0.001, p=0.003, respectively, shown in Table 2).

**Table 2:** – IVUS parameters in UA versus SA patients

Variables	UA(n=43)	SA(n=38)	p
Lesion length, mm	31.77±8.80	31.60±7.86	0.930
MLD, mm	1.47(1.33,1.67)	1.44(1.31,1.67)	0.716
MLA, mm^2^	2.66(2.38,2.95)	2.62(2.09,3.02)	0.936
Plaque area, mm^2^	13.25±2.19	11.06±1.95	0.000
Plaque burden,%	76.66(71.12,81.29)	73.61(69.44,78.77)	0.195
Remodeling index Positive remodeling [n (%)]	0.99±0.15 17(39.5)	0.89±0.12 4(10.5)	0.001 0.003
Negative remodeling [n (%)] Eccentricity index Eccentric lesion [n (%)]	16(37.2) 0.68±0.11 39(90.7)	26(68.4) 0.60±0.13 28(73.7)	0.005 0.003 0.043
Concentric lesion [n (%)]	4(9.3)	10(26.3)	0.043

Continuous variables are presented as means ± SD, median (interquartile range). Categorical variables were expressed as frequencies and percentages. UA: unstable angina; SA: stable angina; MLD: minimum lumen diameter; MLA: minimum lumen area.

### Correlations of legumain concentration with IVUS parameters

The correlation coefficients between legumain concentration and RI in the UA and SA groups were 0.523 and 0.553, respectively (all p<0.001). The correlation coefficients between legumain concentration and EI in the UA and SA groups were 0.486 (p=0.001) and 0.651 (p<0.001), respectively. The above data indicated a significant positive correlation between legumain and RI and between legumain and plaque EI in both the UA and SA groups (shown in Figure 2).

**Figure 2:** – Correlations of legumain concentration with remodeling index (A,B), eccentricity index (C,D) in SA and UA groups. SA: stable angina; UA: unstable angina.

### Multiple logistic regression analysis of cardiovascular risk factors for patients with UA

To investigate cardiovascular risk factors in patients with UA, after including traditional cardiovascular risk factors such as smoking and LDL-C, we conducted multiple logistic regression analyses together with IVUS parameters that differed between the UA and SA groups. The regression analysis showed that the independent cardiovascular risk factors for patients with UA were legumain concentration and coronary plaque area (Table 3).

**Table 3:** – Multiple logistic regression analysis of cardiovascular risk factors for the patients with UA

	Odds ratio	(95%CI)	p value
UA			
Legumain concentration	1.198	1.058-1.356	0.004
Coronary plaque area	1.652	1.150-2.375	0.007

The dependent variable was the presence of UA. The covariate variables included age, gender, hypertension, smoking or not, diabetes mellitus, BMI, homocysteine, LDL-Cl, fasting glucose, triglycerides, total cholesterol, legumain concentration, RI, EI, and plaque area. UA: unstable angina.

### Legumain as a factor for diagnosing UA

ROC analysis was performed to determine the sensitivity and specificity of legumain levels in diagnosing UA. The area under the ROC curve (AUC) of legumain levels for diagnosing UA was 0.789 (95% CI: 0.689-0.888, p<0.001). ROC analysis showed that the optimal cutoff value of the legumain level was 21.68 ng/mL, and the sensitivity and specificity for diagnosing UA were 65.1% and 92.1%, respectively. These results suggested that legumain is highly correlated with unstable lesions. It is expected to be a reliable inflammatory factor for diagnosing CHD, especially UA, to guide early clinical detection of people at high risk for coronary artery lesions(shown in Figure 3).

**Figure 3:** – Receiver operating characteristic (ROC) curves of legumain for diagnosing UA. The area under the curve of legumain was 0.789 (95% confidence interval [CI], 0.689–0.888, p<0.001).

## Discussion

Coronary atherosclerosis is a systemic disease with specific mechanisms that are complex and still not fully understood.^15^ Among them, inflammatory factors play a crucial role in the progression of coronary atherosclerosis and the formation of unstable plaques, which has been confirmed in many previous studies.^16^

As an inflammatory factor, legumain is involved in antigen presentation during atherosclerosis and may induce the formation and progression of unstable plaques, as has been tentatively demonstrated in many studies. As early as 2006, Papaspyridonos et al. identified a significant increase in legumain gene expression within atherosclerotic unstable plaques.^17^ In 2020, Hui Yang et al. investigated the association of legumain with acute myocardial infarction and concluded that legumain is a predictor of all-cause mortality and a potential therapeutic target in acute myocardial infarction.^18^ At the same time, Lunde et al. reached almost the opposite conclusion.^19^ They concluded that legumain is upregulated in acute cardiovascular events and associated with improved outcomes. The reasons for the different results from previous experiments may be as follows: There is a complex interaction between legumain and M1 and M2 macrophages such that the anti-inflammatory and pro-inflammatory effects of macrophages exert different degrees of influence at different stages of the disease and other external factors.^20^ In addition, platelet function is associated with the release of legumain, and a high proportion of patients with cardiovascular disease use antiplatelet drugs such as aspirin, a phenomenon that may also influence the results of the trial.^21^ Our results showed that legumain levels were significantly higher in UA and SA patients than in healthy controls, while legumain concentrations in the UA group were significantly higher than those in SA subjects, suggesting that higher levels of legumain are associated with the development of coronary atherosclerotic disease and the formation of unstable lesions.

In this trial, we found no significant differences in lesion length, MLD, and MLA by comparing the IVUS parameters of coronary lesion vessels in patients in the UA and SA groups. Notably, the plaque area, coronary RI, and plaque EI were significantly higher in the UA group than in the SA group. Moreover, the incidence of positive remodeling and eccentric plaque was significantly higher in the UA group than in the SA group, and the difference was statistically significant. This also indicated that the severity and hazard of CHD are closely related to the instability of the lesion but not necessarily directly related to the stenosis rate of coronary vessels. In general, eccentric plaques are more vulnerable than concentric plaques and are likely to rupture under various intraluminal stresses, which are more often found within unstable lesion segments.^22^ In addition, positive remodeling is widely present within atherosclerotic lesion segments. According to previous IVUS studies, vascular changes in acute coronary syndrome are prone to positive remodeling compared with SA vascular lesions, and positive remodeling is closely associated with adverse events such as plaque rupture and thrombosis.^23^ Additionally, we found that legumain was significantly positively correlated with RI and plaque EI in both the UA and SA groups. In summary, the risk of developing atherosclerotic cardiovascular disease rises in people without CHD as legumain levels increase. In the CHD population, higher legumain levels indicate more severe vascular lesions and more unstable plaques.

Many inflammatory factors are associated with atherosclerosis, but few have high specificity and can be widely used in clinical practice. For example, traditional inflammatory factors such as hs-CRP and homocysteine have many influencing factors, including poor specificity and limited clinical application. The inflammatory factor legumain studied in this trial is highly correlated with coronary atherosclerotic disease and is currently a hot research topic by scholars. The multivariate logistic regression analysis on cardiovascular risk factors in patients with UA in this trial also further suggested that the plasma level of legumain was an independent cardiovascular risk factor in patients with UA (OR=1.198, p=0.004). The AUC for the diagnosis of UA with the legumain level in this study reached 0.789, and the sensitivity and specificity of a legumain level of 21.68 ng/mL for the diagnosis of UA were 65.1% and 92.1%, respectively. The above results suggest legumain’s feasibility, accuracy, and specificity for diagnosing UA. It can be said that legumain is predicted to become an inflammatory factor for the diagnosis of coronary atherosclerotic disease.^24^

Due to the lack of previous studies and reports in the literature on legumain concentration in patients with coronary artery disease and unstable angina, the sample size was determined by a pilot study in this trial. From the pre-experiments, we concluded legumain concentration for the three groups of subjects as 21.18±12.28 ng/ml in the UA group, 14.94±6.14 ng/ml in the SA group, and 8.75±3.01 ng/ml in the control group. The overall sample size was calculated to be 99 cases (33 cases per group) using PASS software, and the final sample size was determined to be 118 cases by considering loss to follow-up and incomplete data.

Our study has several limitations. First, this trial is a single-center, nonrandomized, case-control study with a small sample size, lack of follow-up, and inability to account for causality. Second, the IVUS used in this trial did not have VH-IVUS capability, so it was impossible to classify lesions more finely.^25^ Third, a subgroup of patients with AMI was not established in this trial because performing IVUS prolongs the operation time and may affect the prognosis of the patients. Finally, IVUS has limited resolution in identifying coronary lesions and is less able to identify some specific lesions than OCT.^26^

## Conclusions

High plasma levels of legumain are closely associated with the development of coronary atherosclerotic disease and the progression of unstable lesions. Detecting plasma legumain can help identify patients at high risk of CHD earlier so that intervention may occur earlier and the prognosis may be improved.
